# The impact of neck tilt on the accuracy of deep learning generated contours for CT images of the head and neck

**DOI:** 10.1002/acm2.70316

**Published:** 2025-11-03

**Authors:** Jamison Brooks, William Harmsen, David Routman, Erik Tryggestad, Douglas Moseley

**Affiliations:** ^1^ Department of Radiation Oncology Mayo Clinic Rochester Rochester Minnesota USA; ^2^ Department of Quantitative Health Sciences Mayo Clinic Rochester Minnesota USA

**Keywords:** Deep learning autosegmentation, head and neck radiotherapy, organs at risk, patient positioning, quality assurance

## Abstract

**Background:**

Deep learning auto segmentation (DLAS) tools are widely used for radiation therapy planning, yet limited information exists on how patient positioning impacts their performance, particularly in head and neck (HN) computed tomography (CT) imaging.

**Purpose:**

This study investigates the impact of abnormal neck tilt on the accuracy of DLAS‐generated contours in HN CT scans. We hypothesize that abnormal positioning degrades auto segmentation performance, particularly for organs at risk (OARs) influenced by cervical spine orientation.

**Methods:**

A total of 35 HN CT scans were retrospectively analyzed. Neck tilt was quantified using principal component analysis of the spinal cord contours from C1 to C4, with patients stratified into normal and abnormal tilt groups based on percentile thresholds. Seven FDA‐cleared DLAS tools were evaluated for OAR segmentation accuracy across brainstem, parotid glands, submandibular glands, brachial plexus, and optic nerves. Gold‐standard contours were curated from manually created clinical contours and compared to DLAS output using Sorenson's volumetric dice similarity coefficient (DSC), surface Dice similarity coefficient with a 2 mm threshold (sDSC), mean distance to agreement (MDA) and difference in mean dose. Statistical differences were assessed using Wilcoxon rank‐sum tests (*p* < 0.05).

**Results:**

Among the evaluated OARs, the parotid glands showed consistent and statistically significant degradation in contouring accuracy for patients with abnormal neck tilt across all metrics (DSC, sDSC, MDA) and across all seven DLAS tools. Changes in contouring accuracy resulted in significant differences in both absolute and signed difference in mean dose. No consistent differences were observed for other structures across multiple DLAS tools.

**Conclusions:**

Abnormal neck tilt is associated with reduced DLAS accuracy and greater dose variability for parotid gland segmentation in HN radiotherapy planning. These findings underscore the need for enhanced patient quality assurance strategies for patients with abnormal positioning when using clinical DLAS workflows. Future work should explore automated detection of anatomical outliers and site‐specific model retraining to ensure accurate contouring delineation for patients with atypical positioning or anatomy.

## 1 INTRODUCTION

Recent advancements in deep learning auto segmentation (DLAS) have resulted in many commercially available models for the analysis of Computed tomography (CT) scans of human anatomy.^1^, ^2^, ^3^ DLAS models have demonstrated the ability to produce expert level contours which both saves time and reduces inter‐observer variability compared to manual contouring.^4^, ^5^ Despite the many commercially available models, relatively little information is available regarding the conditions under which these tools are not reliable for head and neck anatomy.

In head and neck (HN) patients, anatomical variation can arise from multiple factors, including gross tumor burden, prior radiation therapy, surgical resection, dental hardware, and reconstructive procedures. Patient positioning during simulation—particularly neck tilt—can also introduce significant variability. Patients are positioned to be reproducible, stable, and comfortable for the duration of their treatment by adjusting the amount of head cushioning and immobilization on a patient‐to‐patient basis. For most patients this results in a head position with minimal neck tilt (no extensive neck flexion or extension). However in some cases, excessive neck tilt may result from suboptimal setup or spinal conditions such as kyphosis. Prior work with atlas‐based auto segmentation has demonstrated that segmentation accuracy is sensitive to differences in neck tilt between the patient CT and the reference atlases.^6^ However, much less is known about how such positioning deviations affect the performance of DLAS algorithms. Based on our clinical experience, we hypothesize that abnormal positioning—such as moderate to severe neck tilt—similarly reduces contouring accuracy in DLAS models.

A barrier to investigating the impact of neck tilt on DLAS performance is the challenge of quantifying neck tilt. While several methods exist for measuring head tilt,^7^, ^8^ these primarily assess cranial orientation and do not account for cervical spine curvature, which may be equally or more relevant in the context of HN radiotherapy planning. Evaluating the orientation of the upper cervical spine offers a more comprehensive metric by capturing both head tilt and neck curvature. This approach may better reflect the true treatment setup and may be more sensitive for detecting clinically relevant postural deviations, such as those seen in patients with kyphosis. However, because the vertebra of the cervical spine tilt in relation to one another, it may be a more complicated measurement than estimating head tilt.

In this work, we develop an approach to quantify neck tilt in the sagittal plane using DLAS of the cervical spine. Using this approach, we stratify patients into groups with typical and abnormal neck tilt to identify its impact on the quality of contouring from seven FDA approved user ready DLAS tools. To assess contour accuracy, Sorenson's volumetric dice coefficient (DSC), mean distance to agreement (MDA), and surface Dice similarity coefficient (sDSC) are used to compare contours to a high‐quality set of manually derived contours to assess contour quality. Following the geometric analysis, difference in mean dose was calculated to quantify dosimetric differences between DLAS‐generated and gold‐standard contours using clinically delivered treatment plans.

## 2 METHODS

### 2.1 Patient data curation and DLAS models

Patient data for this study consisted of retrospectively collected data from 35 patients with HN cancers who underwent radiotherapy at Mayo Clinic Rochester (Rochester, MN, USA) and Mayo Clinic Arizona (Scottsdale, AZ, USA). The use of retrospective HN patient data for model training was deemed exempt by IRB. The dataset contained a range of HN disease sites and progressions, including patients with prior resection, and represented the current treatment landscape at the institutions. CT images were acquired at simulation before the start of radiotherapy treatment using multiple Somatom Definition AS (Siemens, Munich Germany) CT scanners with voxel dimensions of 1.27 mm × 1.27 mm × 2 mm. The CT images were acquired at 120 kVp and most were reconstructed using iterative metal artifact reconstruction techniques to minimize artifacts caused by dental fillings or other metallic objects commonly present during HN radiotherapy.

Contours for the brain stem, parotid glands, submandibular glands, brachial plexuses and optic nerves were created both manually and with seven pretrained commercially available DLAS models. Contour types were chosen based on their availability for DLAS auto segmentation models, their relevance as OAR's in radiation therapy, and their likelihood to be perturbed by changes in neck tilt.

Manually delineated clinical contours from the 35 patients were reviewed and edited by Radiation Oncologists specializing in treatment of the head and neck. A thorough description of the contour curation efforts has been previously published.^9^ Importantly, this manual contour curation process occurred outside of the normal clinical time constraints; consequently, the quality and consistency of resulting contouring and editing is ideal. The resulting curated, that is, “gold standard,” contours were used as a reference for comparison to contours from DLAS tools.

### 2.2 Neck tilt calculations

Neck tilt calculations were performed by first segmenting the vertebral bodies and spinal cord. This was performed with the open source DLAS tool TotalSegmentator and evaluated by a board‐certified medical physicist.^10^ Following from this output, the longitudinal extent of the cord contour overlapping with the first through fourth cervical spine is obtained. Principal component analysis was performed on the coordinates of the voxels contained in the cord contour. The Y and Z components of the first principal component vector are obtained and the angle between this vector and the longitudinal (Z) axis are calculated as the neck tilt angle.

To ensure the accuracy of the PCA based neck tilt calculations, the slope of a linear best fit line through the C1–C4 centroid positions in the sagittal plane was compared with the PCA‐derived neck tilt angles. The centroid positions were well described by the linear fit, and the slopes showed strong linear correlation with the PCA‐based measurements. This agreement indicates that the PCA‐derived neck tilt angle provides an accurate representation of upper cervical spine positioning (data not shown).

An upper and lower limit on neck tilt angle were identified to set the cutoff for normal neck tilt angle. Normal neck tilt was defined as falling between the 12th and the 88th percentile. This range was determined by a board‐certified medical physicist by identifying a cutoff that included 8 abnormal patients (for reasonable statistical power) and then evaluating the range of neck tilt values and the corresponding CT images in the 35 patient cohort. This method aimed to balance statistical power and separation of tilt values in the outlier cohort from the normal cohort. The total segmentator DLAS model was not one of the seven DLAS models evaluated in this study.

### 2.3 Contour analysis and results preparation

Comparison of contours from each DLAS tool to the gold standard contours was performed using sDSC with a 2 mm tolerance,^4^ DSC and MDA^11^ using MATLAB (version 2023b, MathWorks, Natick, Massachusetts). Absolute and signed difference in mean dose was calculated by taking the difference in mean dose for gold standard and DLAS contours using existing patients’ existing clinical treatment plans to assess the dosimetric impact of geometric differences. To ensure adequate dose for dose analysis, patients with gold standard OARs that received a mean dose less than 5 Gy were excluded from the difference in mean dose analysis. A two tailed Wilcoxon rank‐sum test with *p*‐value of 0.05 was used to determine whether the distributions of all metrics and doses differed significantly between normal and abnormal neck tilt cohorts.

## 3 RESULTS

**FIGURE 1:**
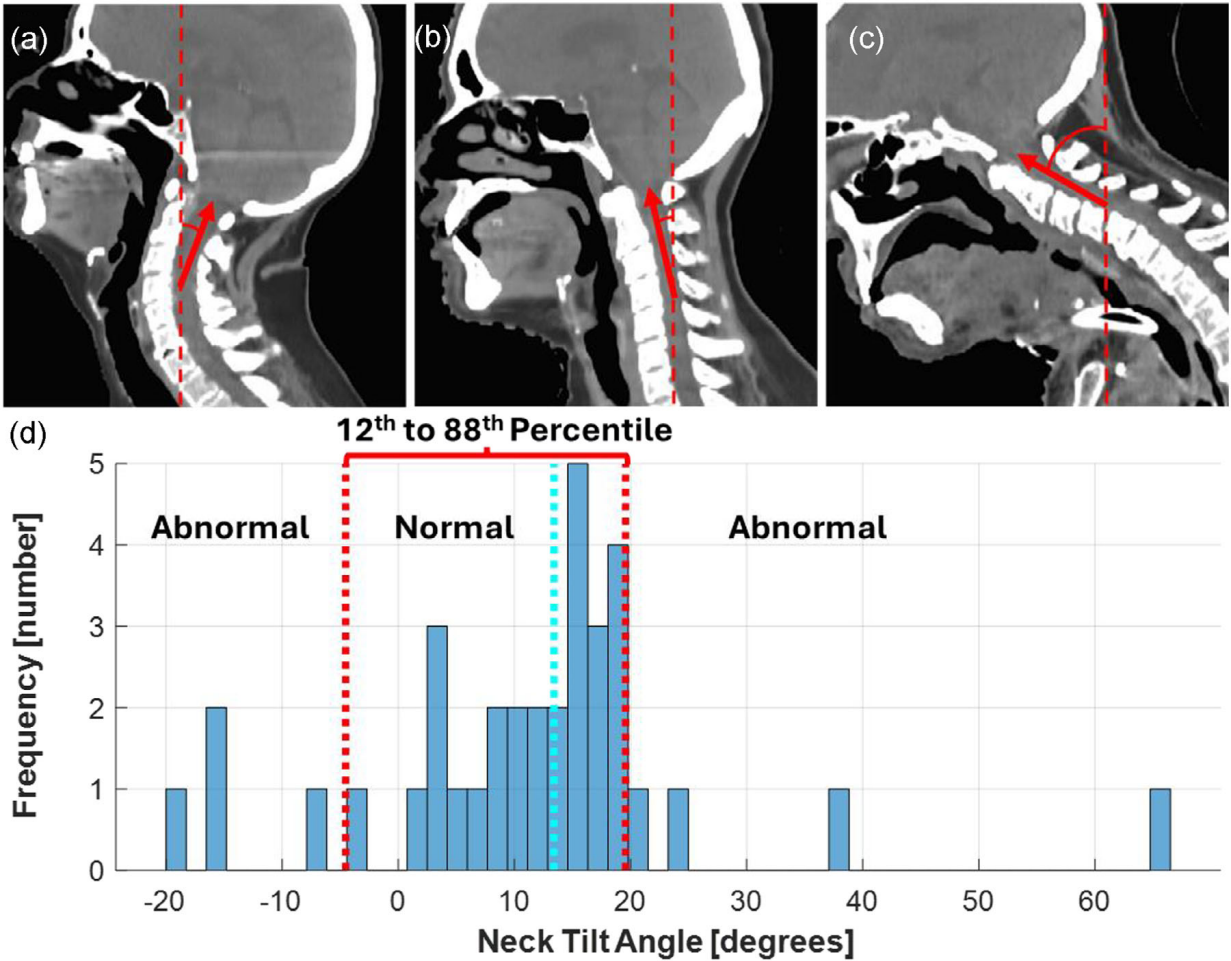
CT images are shown with −19.5° abnormal, 15° normal, and 66° abnormal neck tilt (a–c respectively). A histogram displaying the range of neck tilts is shown (d). The red lines indicate the lower and upper 12th and 88th percentile cutoffs (corresponding to −5 and 20°) used to classify normal and abnormal neck tilt. The median is displayed with a cyan line.

**FIGURE 2:**
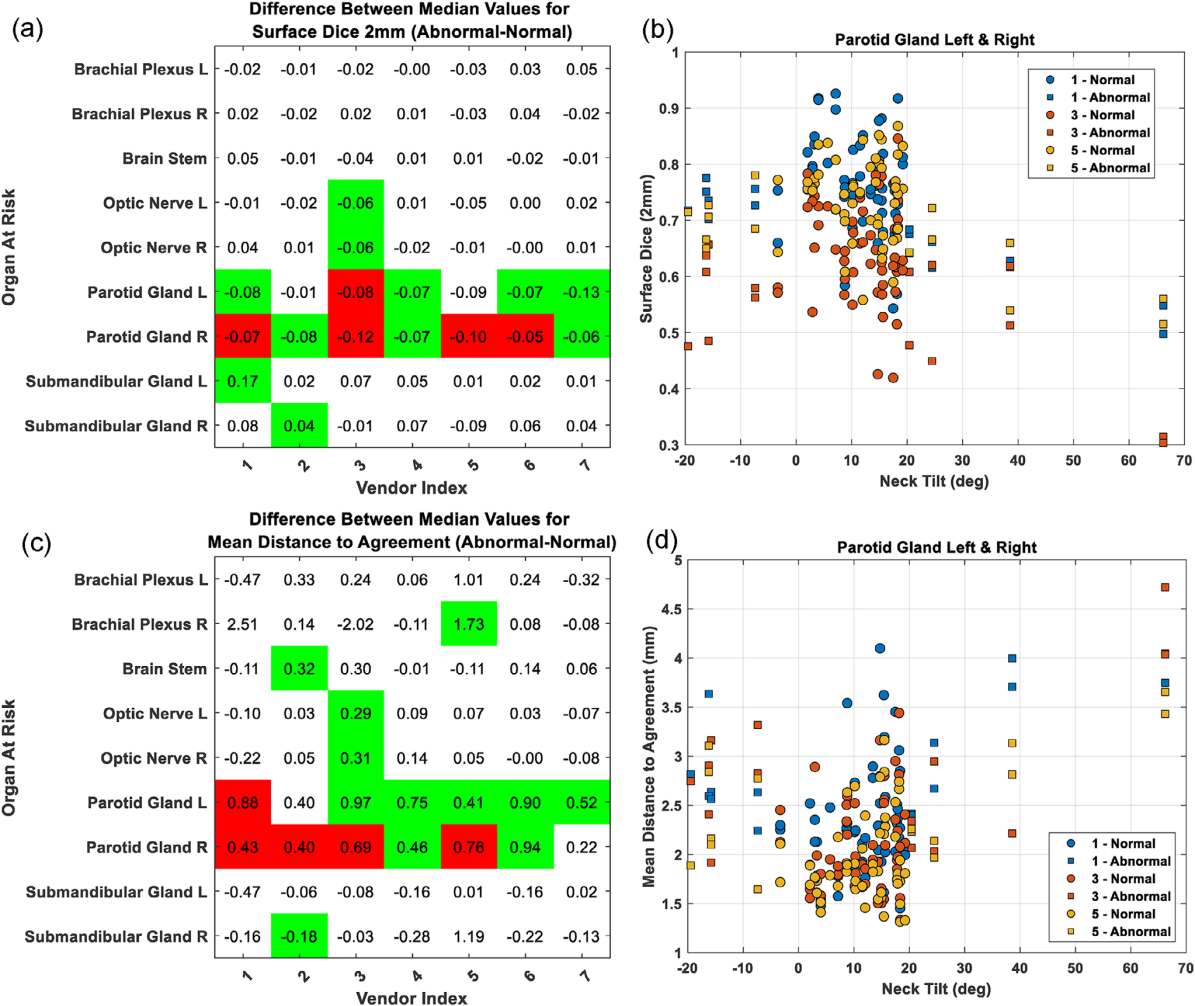
Difference in sSDC (a) and MDA (c) between abnormal and normal neck tilt groups for seven different commercially available DLAS tools. Metrics were calculated by comparing each DLAS based contour to a high‐quality manually delineated gold standard contour. Green indicates significance at a value of *p* < 0.05 and red indicates significance at a level of *p* < 0.01. Scatter plots showing neck tilt versus sDSC (b) and MDA (d) for abnormal and normal left and right parotid glands are shown for vendors 1, 3, and 5. Similar trends in decreased sSDC and increased MDA are observed as neck tilt deviates more from the median across vendors.

The separation of patients into normal and abnormal neck tilt is shown in Figure 1. The normal neck tilt range was approximately −5° and 20°, with negative and positive values indicating extension and flexion, respectively. Differences in median values between normal and abnormal neck tilt using sDSC, and MDA metrics across multiple commercially available DLAS tools and OARs are shown in Figure 2a,c. A corresponding plot for DSC is shown in the supplement (Figure S1A). Consistent statistically significant differences in sDSC, DSC, and MDA (*p* < 0.05) are observed for the left and right parotid glands. Other OARs did not show consistent differences between CT scans of patients positioned with normal and abnormal neck tilt across commercially available DLAS tools.

Scatterplots of neck tilt versus contour quality metrics for left and right parotid glands are shown in Figures 2b,d and S1B. A trend of increased contour variation can be seen for patients with abnormal variations in neck tilt across different vendors. Similar patterns were seen between left and right parotid glands (Figure S2).

**FIGURE 3:**
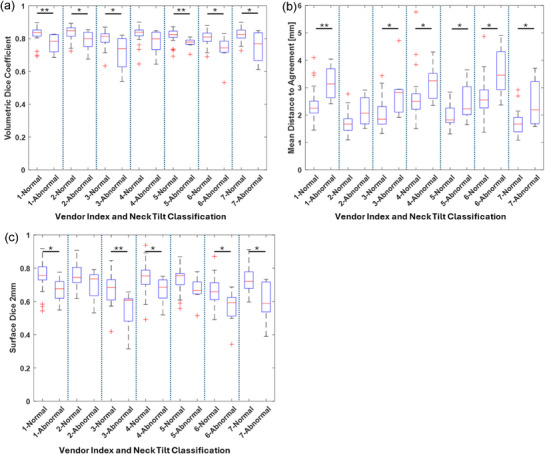
Box and whisker plots for (a) the DSC, (b) MDA and (c) sDSC for the left parotid gland. Comparisons between normal and abnormal neck tilt are shown for each commercially available DLAS tool (* indicates *p* < 0.05, ** indicates *p* < 0.01).

**FIGURE 4:**
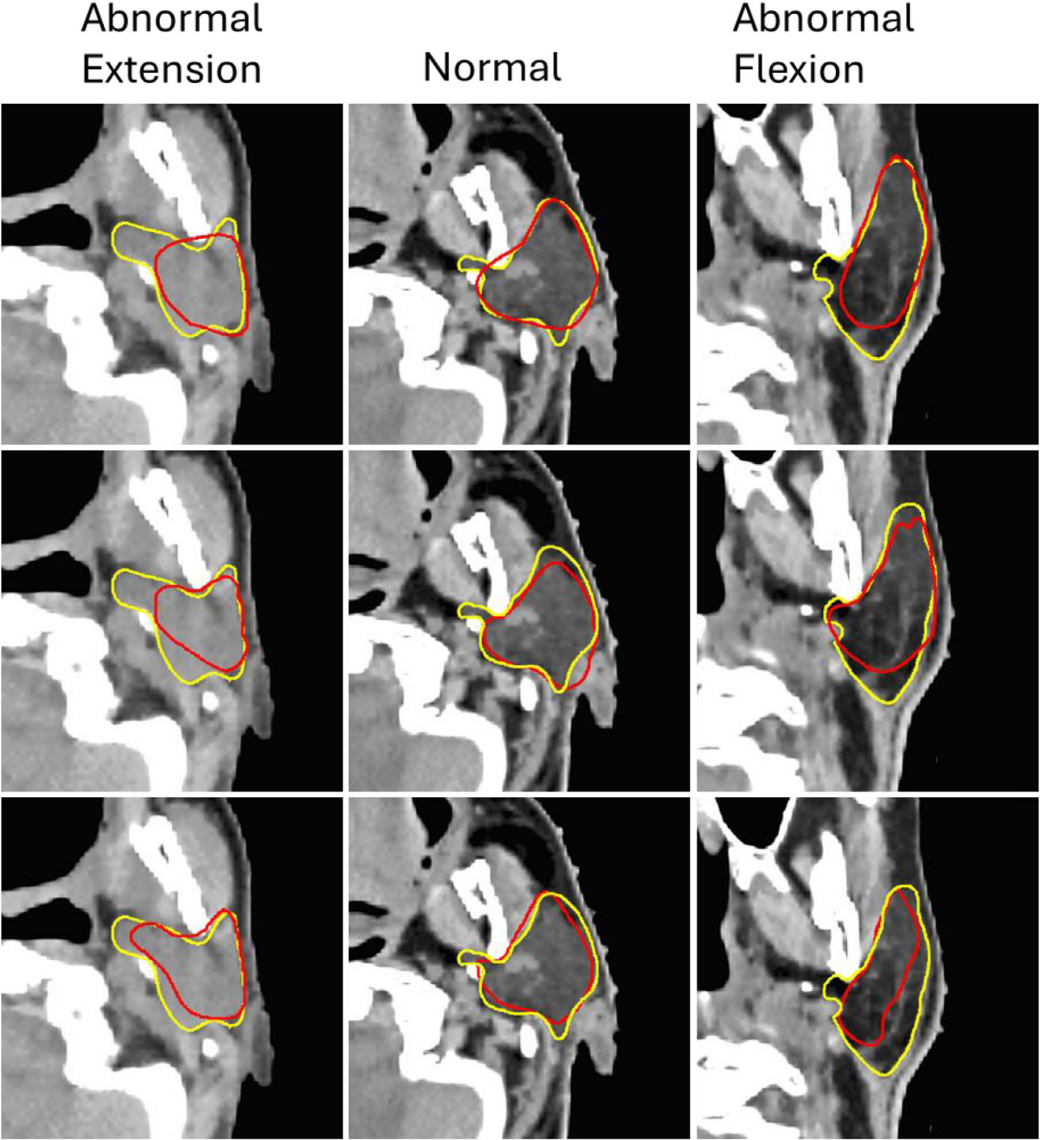
CT images with gold standard parotid reference contours (yellow) and vendor reference contours (red) for vendors 5, 7, and 3 (top to bottom) are shown. Neck angles are −16°, 11.5°, and 66° (left to right).

Box and whisker plots of contour metrics for normal and abnormal head tilt groups are shown in Figures 3 and S3, highlighting better agreement between gold‐standard parotid gland contours and DLAS‐generated contours in the normal neck tilt cohort. Visual examples of increased variation in DLAS parotid gland contours compared to gold standard contours are shown in Figure 4.

**FIGURE 5:**
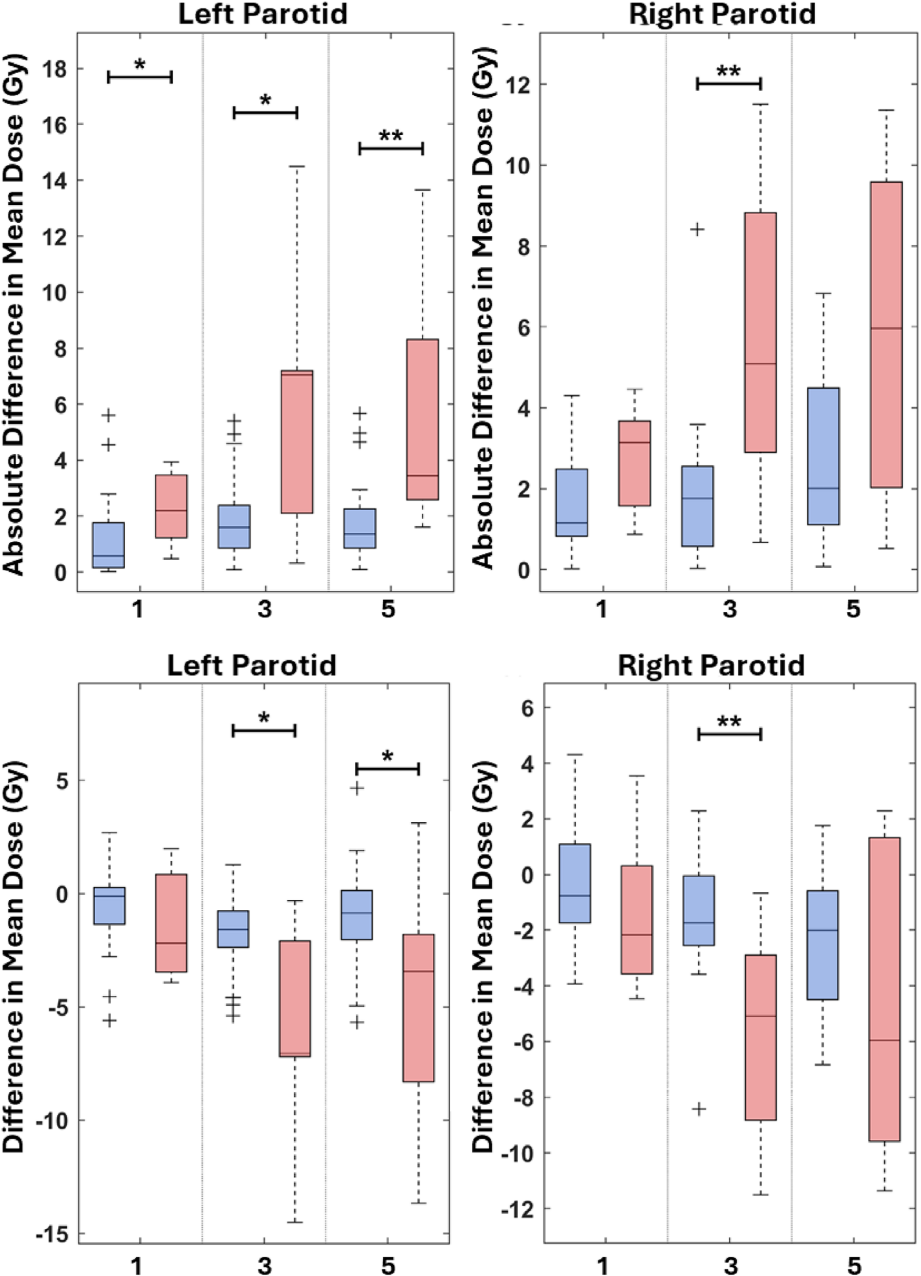
Absolute and signed difference in mean dose between gold‐standard and DLAS‐generated parotid gland contours for vendors 1, 3, and 5. Top row: Absolute difference in mean dose (Gy) for left (left panel) and right (right panel) parotid glands. Bottom row: Signed difference in mean dose (Gy) for left (left panel) and right (right panel) parotid glands. Blue boxplots represent the normal neck tilt cohort, and red boxplots represent the abnormal neck tilt cohort. Boxes indicate the median, and interquartile ranges and whiskers denote the range excluding outliers. Statistically significant differences between groups are indicated by asterisks (* indicates *p* < 0.05, ** indicates *p* < 0.01). The number of contours in each group were 26 normal and seven abnormal left parotid glands and 25 normal and seven abnormal right parotid glands after excluding patients with gold standard contours receiving less than 5 Gy mean dose. Abnormal neck tilt was associated with larger absolute dose deviations and greater variability in both absolute and signed difference in mean dose compared to normal neck tilt.

Following the geometric analysis, difference in mean dose between gold standard and DLAS parotid gland contours was calculated for vendors 1, 3, and 5 to assess the clinical relevance of the dose changes. Vendors 1, 3, and 5 were chosen as they exhibited consistent differences in geometric metrics. Significant differences in both absolute and signed difference in mean dose were observed for right and left parotid glands (Figure 5). Increased median values of absolute difference in mean dose are observed for all three vendors for both left and right parotid.

## 4 DISCUSSION

The findings of this study support the hypothesis that abnormal neck tilt is associated with reduced performance of DLAS tools for OARs in head and neck CT imaging. Among the OARs evaluated, the parotid glands demonstrated the most consistent and statistically significant differences in geometric contouring accuracy across multiple DLAS tools between normal and abnormal neck tilt groups. Abnormal neck tilt also led to greater differences in mean parotid dose for DLAS‐generated contours compared to gold standard contours—a clinically relevant dosimetric metric in head and neck radiotherapy. These findings highlight the importance of understanding anatomical variation and patient setup when deploying DLAS in clinical workflows.

To our knowledge, this is the first study to quantitatively assess the impact of moderate to severe head tilt on the performance of commercially available DLAS tools. While understanding the appropriate use cases for DLAS models is essential, limited research has examined their failure modes. One prior study evaluating three commercial DLAS tools in prostate cancer patients reported significantly reduced performance in cases with anatomical outliers compared to anatomically typical cases.^12^ Additional studies have also identified abnormal anatomy—either qualitatively or quantitatively—as a factor contributing to reduced contouring accuracy in DLAS models.^13^, ^14^ These findings align with the results of our study, which demonstrate that model performance degrades in the presence of anatomical deviations.

A critical factor influencing DLAS performance is the nature of the training data. Variability in image parameters, patient anatomy, and patient positioning all contribute to the generalizability of a model. If training datasets underrepresent cases with abnormal anatomy or positioning, the model may learn a biased representation of typical anatomy and fail to generalize to real‐world clinical diversity. This is especially relevant in head and neck imaging, where surgical modifications, hardware artifacts, and neck tilt are relatively common. To mitigate this, data augmentation strategies and diversity in training cohorts are essential for improving robustness. Traditional augmentation techniques, such as rotation, scaling, and intensity variations, have been widely employed to increase data diversity and improve model performance.^15^ However, these conventional methods may not sufficiently capture the complex anatomical variations present in outlier cases. Recent advancements in deep generative models, including generative adversarial networks (GANs) and variational autoencoders (VAEs), have shown promise in generating realistic and diverse medical images that reflect rare or abnormal anatomical structures. By incorporating these synthetically generated examples into the training dataset, DLAS models can be exposed to a broader spectrum of anatomical variations, thereby improving their generalization capabilities and performance on uncommon or complex cases.^16^

In addition to augmentation, clinical retraining of DLAS tools using site‐specific datasets—including anatomically diverse or previously incorrectly contoured cases—has been shown to enhance local model performance.^17^, ^18^ This approach allows models to learn from institution‐specific imaging protocols and patient populations, potentially correcting for biases introduced during initial training on more generalized datasets.

One additional explanation for the variability in DLAS performance is the complexity of the underlying model architecture. While specific model architectures were not disclosed for the commercial DLAS models analyzed here, differences in performance may stem from whether a model is based on two‐dimensional (2D) slice‐by‐slice, non‐contiguous slice, or fully three‐dimensional (3D) volumetric inferencing. It has been demonstrated that 3D models often achieve higher segmentation accuracy compared to their 2D counterparts.^19^ However, 3D models also require greater computational resources. The inability of some tools to adapt to abnormal neck curvature may reflect limitations in model design.

The parotid glands appear to be more susceptible to positional changes introduced by neck tilt. This may be due to their anatomical location lateral to the upper cervical spine, where flexion or extension can substantially displace the glands relative to fixed bony landmarks. In contrast, structures such as the brainstem and optic nerves are more rigidly anchored and may be less affected by changes in patient positioning.

Another contributing factor to reduced DLAS accuracy in cases with abnormal neck tilt may stem from the difficulty of manual contouring in these anatomically atypical patients. Clinical observers are often trained and experienced with normal anatomy, and their consistency may degrade in cases that deviate from the typical presentation. As a result, the manually defined contours used as reference standards in this study may carry higher interobserver variability in patients with severe neck tilt. This inherent variability in the reference contours could partially account for the observed reduction in measured DLAS performance. Incorporating additional expert or consensus contours in future work may help isolate DLAS performance from human variability to better clarify the source of segmentation discrepancies.

This study has several limitations. First, the cohort size was relatively small, limiting statistical power, especially for OARs with smaller volume or less anatomical variability. Second, while neck tilt provides a quantifiable and interpretable metric for patient positioning, it may not capture other forms of anatomical abnormality that also affect contouring accuracy, such as post‐surgical changes or the presence of tumors. Additionally, our neck tilt measurement, based on principal component analysis of the spinal cord, while robust, may be less applicable to evaluating structures further from the upper C‐spine regions.

Future work should focus on expanding the patient cohort to encompass a broader range of anatomical presentations, surgical histories, and imaging protocols and develop ways to mitigate the influences of such outliers on DLAS. It would also be valuable to investigate alternative or complementary approaches for identifying patient abnormalities, such as machine learning classifiers trained to detect atypical anatomy or positioning. Although manual review by clinical staff is required and remains the gold standard for evaluating DLAS output, it is time‐intensive and subject to interobserver variability. Recent studies have proposed augmenting manual contour review with automated contour evaluation models.^20^, ^21^, ^22^ When combined with models designed to flag abnormal patient anatomy or positioning, these tools may improve error detection rates and reduce the burden of manual review. A potential workflow could involve pre‐screening scans for anatomical outliers, followed by an automated assessment of DLAS‐generated contours to assist clinical reviewers in identifying problematic cases more efficiently. These findings underscore the importance of considering patient‐specific anatomical factors when evaluating and deploying DLAS tools in clinical practice. Improving provider awareness to failure modes and model robustness to variation is essential for ensuring consistent, high‐quality care across diverse patient populations.

## 5 CONCLUSION

In conclusion, our findings highlight that neck tilt significantly impacts the performance of some commercially available DLAS tools in contouring head and neck anatomy, particularly for the parotid glands. Further development and validation of DLAS models using diverse datasets are necessary to ensure robust performance across a range of anatomical presentations. Implementing strategies to detect or adjust for abnormal neck tilt and potentially other abnormal anatomy in clinical workflows has the potential to enhance the accuracy and reliability of automated contouring, ultimately contributing to improved radiation therapy planning and patient care.

## AUTHOR CONTRIBUTIONS

The manuscript was written by Jamison Brooks. Dataset curation and analysis was primarily performed by Jamison Brooks, William Harmsen, Erik Tryggestad, and Douglas Moseley. Manuscript conception, design, and editing was performed by Jamison Brooks, Douglas Moseley, Erik Tryggestad, William Harmsen, and David Routman. All authors approved the submitted article.

## CONFLICT OF INTEREST STATEMENT

The authors have no relevant conflicts of interest to disclose.

## ETHICS STATEMENT

The use of the retrospective patient data in this work was deemed exempt by IRB.

## Supporting information



Supporting Information

## Data Availability

The authors cannot share the data at the time of publication. Please contact the authors for additional information.
