# Supplementary material for: The impact of neck tilt on the accuracy of deep learning generated contours for CT images of the head and neck
Source: J Appl Clin Med Phys. 2025 Nov 3;26(11):e70316. doi: 10.1002/acm2.70316 (PMC12582642; doi:10.1002/acm2.70316)
Supplement: Supplementary file 1 — Supporting Information [file ACM2-26-e70316-s001.docx]

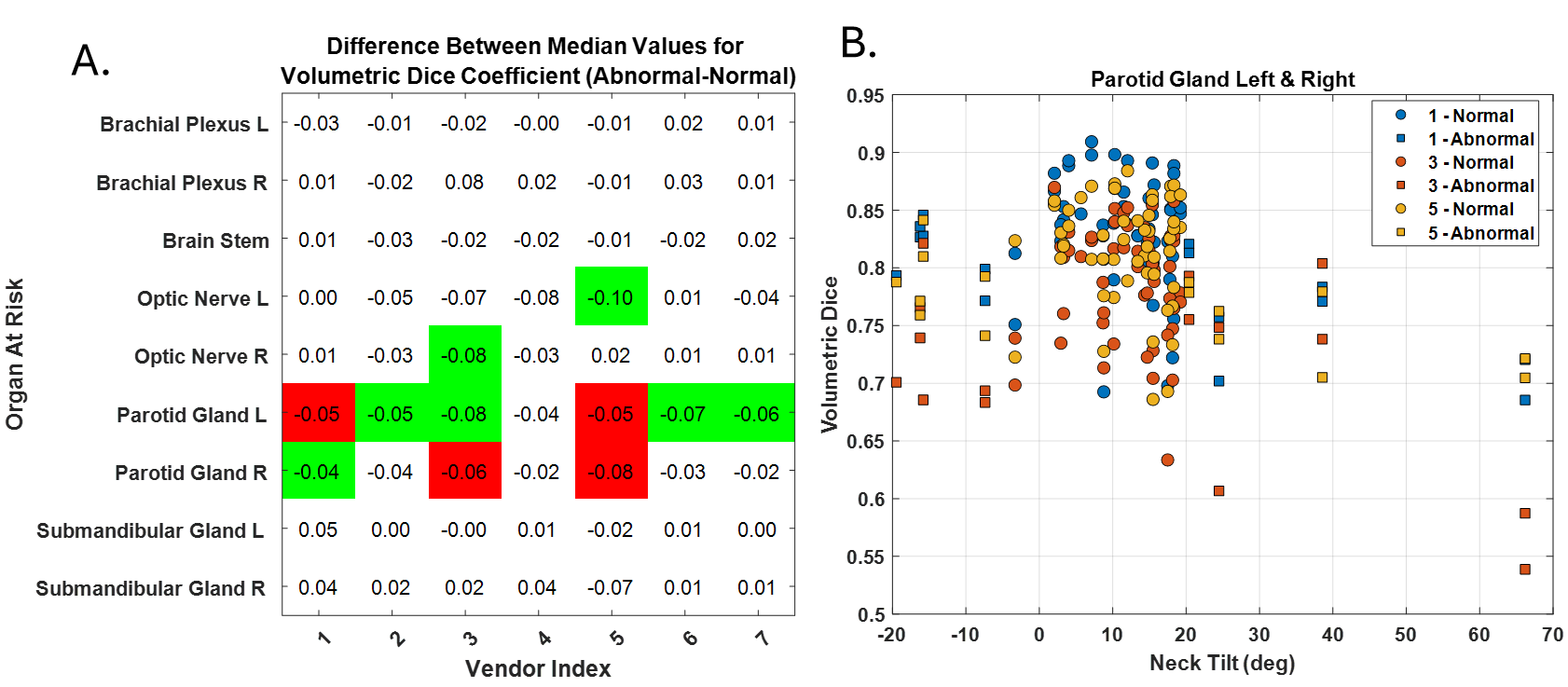


**Supplemental Figure 1:** Difference in median volumetric Dice coefficients between abnormal and normal neck tilt groups for seven different commercially available DLAS tools (A). Volumetric Dice coefficient values were calculated comparing each DLAS based contour to a high-quality manually delineated gold standard contour. Green indicates significance at a value of p<0.05 and red indicates significance at a level of p<0.01. Scatter plot showing the effects of neck tilt on Sorenson’s Volumetric Dice coefficient (B).

**
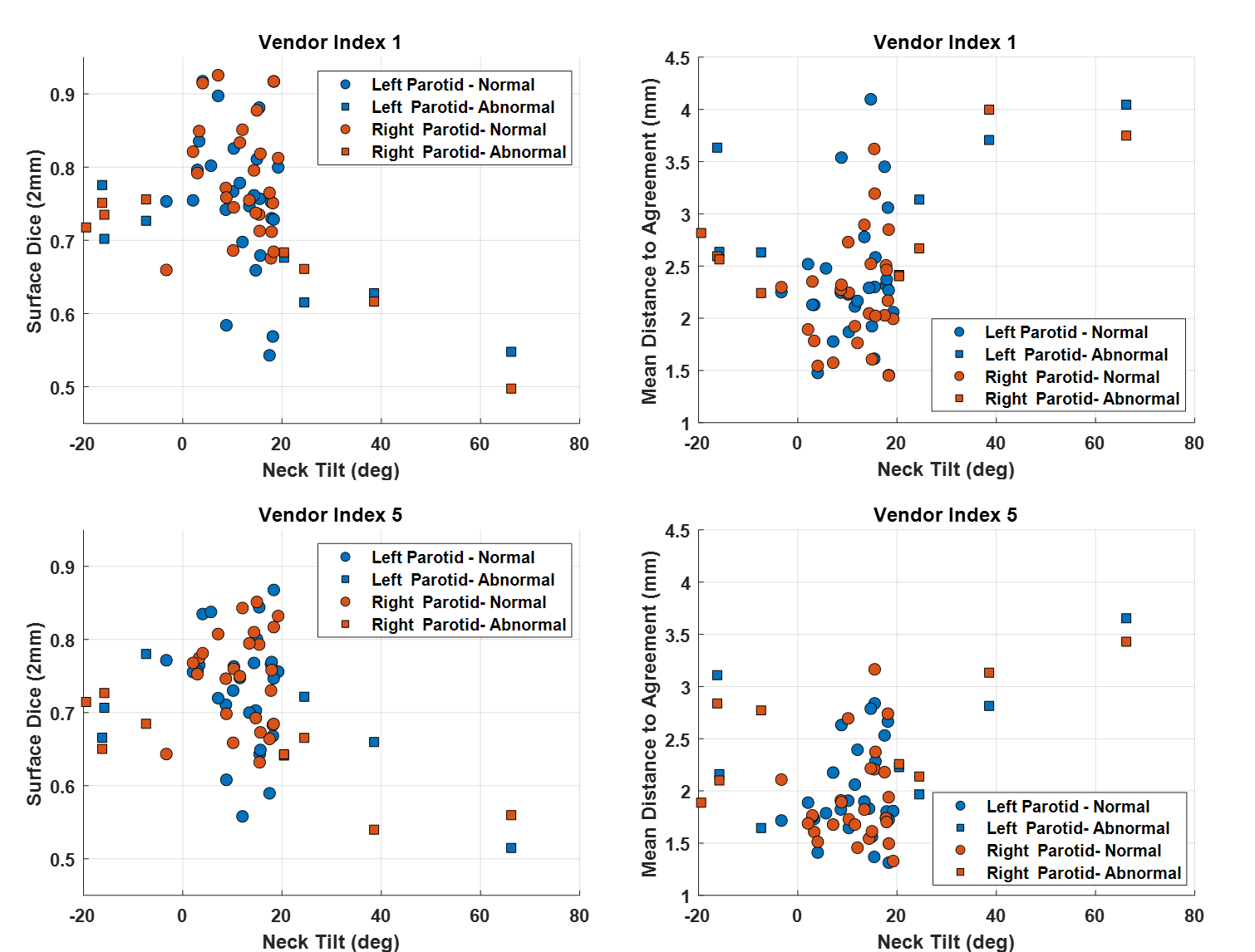
**

**Supplemental Figure 2.** Scatterplots of surface Dice (2 mm) and mean distance to agreement (MDA) versus neck tilt for DLAS tools from vendors 1 (top) and 5 (bottom). Blue = left parotid, red = right parotid; circles = normal neck tilt, squares = abnormal neck tilt.

**
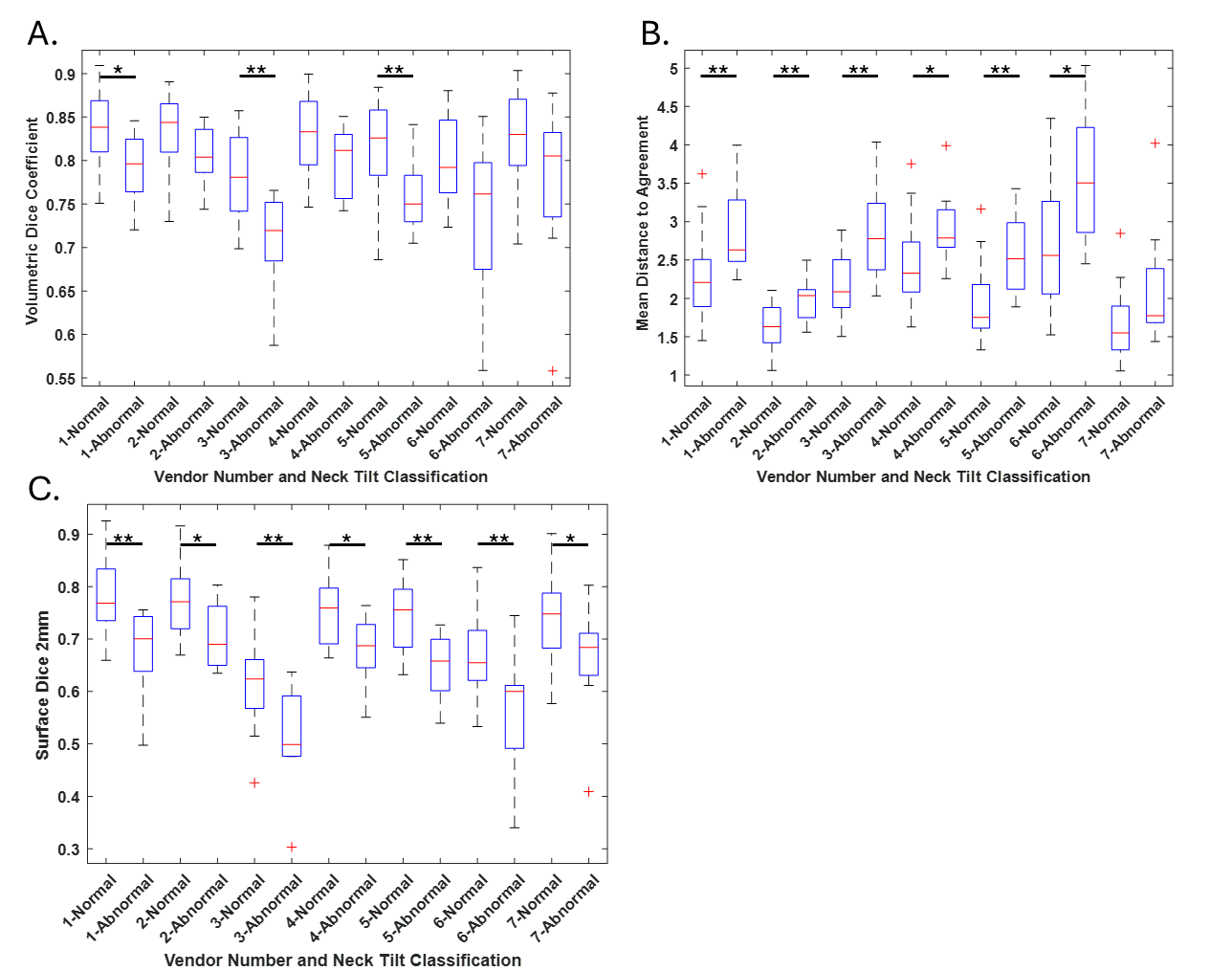
**

**Supplemental Figure 3:** Box and whisker plots for (A) the volumetric Dice coefficient, (B) mean distance to agreement and (C) surface Dice with a 2mm threshold for the right parotid gland. Comparisons between normal and abnormal neck tilt are shown for each commercially available DLAS tool (* indicates p<0.05, ** indicates p<0.01).
